# Supplementary material for: Benchmarking of an Intervention Aiming at the Micro-Elimination of Hepatitis C in Vulnerable Populations in Perpignan, France, to Inform Scale-Up and Elimination on the French Territory
Source: Viruses. 2024 Oct 21;16(10):1645. doi: 10.3390/v16101645 (PMC11512308; doi:10.3390/v16101645)
Supplement: Supplementary file 1 [file viruses-16-01645-s001.zip › viruses-3174145-supplementary.pdf]

Table S1- Qualitative patient interviews- main themes and quotes.

**Specialised staff encouraged patient engagement and retention with *Test & Treat* programme. -**Participants mentioned that staff always followed-up with them post-treatment. They felt taken care of, without facing judgement (assistance with paperwork etc.) Customized care, not being treated "like a number." Programme staff reminded patients of their next hospital appointment.
**Interview 1: "***I was accompanied every time there and there was a nurse to help me, because I can't read or write well, and she helped me with the papers for the services, and when I called her, she was always there for me. She was waiting for me at the door, and she took me everywhere. Yes, that's what I thought was so good because it was so organised. And it should be done more in other countries, so it would be good."* **Interview 2:** *"thanks to them I was able to have the treatment and be cured, I thank them very much. "* **Interview 3:** *"They were always checking up on me, seeing how I was doing, helping me with my medication. I was really helped."* **Interview 6:** *"They've picked me up by car, they've driven me back, 3 or 4 times. They have been pleasant, very nice, they give their all, they care about it, you can see it, you can feel it."* **Interview 9:** *"They would remind me of my appointments. They followed up on me, reminded me of my blood tests."* **Interview 10:** *"Because they are present. I'm not a number. They are really good people."* **Interview 11: "***It's a well-coordinated team that surrounds you. That's what's good." "we've never seen anything like it in Perpignan, and what's more, in a neighbourhood where there are gypsies, who, by using, by using, they are sick. They phone me once, they phone me twice, they even pass on words to the nurses who work with us, who give injections, they ask the nurses to tell us to go to the hospital to get the prescription."* **Interview 12:** *"So, we need people like these little angels, who go from village to village to save people. I can never thank them enough for what they have done. For me, it's huge. Because in a way they saved my life. I'm ready to continue with them wherever they want to go." "The programme team was great, they didn't let me down."*<br>*"They told me everything and were frank from the start. That gives you confidence. Because, at my age, I didn't know where I was going."* **Interview 13:** When questioned about being able to access treatment without this Test & Treat programme: *"No, I think not, because I don't have enough money. They facilitated financially."* **Interview 14:** *"because of their sympathy, their interest in me, their way of supporting me, as soon as there was a problem, they gave me their telephone numbers, they could be reached, things like that. These are things that a doctor won't do."*
**Access to new DAA treatment enabled engagement with treatment and treatment completion**. Access to treatment was convenient/flexible (e.g., home delivery). Oral medication was preferred among patients who had previously tried the old treatment or heard about the old treatment's low success rate. Little side effects and high chance of treatment success intrigued patients.
**Interview 1:** *"I took my medication, there was one a day to take, I took it, it didn't hurt me. It didn't hurt, it didn't hurt at all."* **Interview 2:** *"It was knowing that I was going to heal mostly and they could make the delivery directly to the house without anyone knowing. "* **Interview 3:** *"We will say that there are no side effects, it's not a treatment that is difficult, if you take the medication every day, that's that."* **Interview 5:** *"I have never tried the old treatments, but I have heard a lot about it, but apparently it was still a pretty tough treatment. But that's also what made my decision easier, for sure the treatment we have now is easier than the old ones. Nothing to improve."* **Interview 8:** *"It doesn't do anything, there are just some pills to take."; "it was a pleasure. Especially since it's only pills, tablets, to take for Hepatitis C. "* **Interview 9:** *"The best thing was, it's fine, it was just capsules to take, and that was great, no injections and all that."* **Interview 10:** *"Well, it was great, the doctor told me I was ill, he said "Do you want to be treated? "and I said yes and the same day I started the treatment."* **Interview 11:** *"And when I talked with my colleagues, I saw the bus, I said how it works and all that, it's convenient, I mean, we are comfortable. They said "do you think we can go there? "I said "we can go, it's not interferon injections anymore, it's pills, I took them, and thanks to God, I'm cured of it."* **Interview 12:** *"the treatment was flawless. I didn't feel any more pain or fatigue than usual. This fatigue was normal."* **Interview 14:** *"Yes, that's what I was treated with, not the old treatment which was a bit heavy."*

**Receiving HCV treatment led to additional positive outcomes for patients.** Patients stopped using drugs and alcohol and cut ties with negative (influential) social groups. Participants were eager to be good a parent/spouse and look after themselves to be there for their children/families/partner and improve their life.
**Interview 1:** *"No, I stopped, now I don't drink anymore. Since I started the treatment in October/November last year, I don't drink anymore." "I listened and then it gave me courage to go further and live for my children and not leave them very young."* **Interview 2: "**To take care of myself because I have two children and that's my whole life. *"; "to finally be able to heal and no longer have injection problems. " "I learnt a lot and it allowed me to think much better, to move forward much better. Thanks to this I was able to stop the codeine."* **Interview 3:** *"since I don't drink anymore my liver will regenerate and I am happy about that."* ***"****I don't drink any more, I don't do drugs anymore, I am really very lucky. And I got my wife back, we cancelled the divorce, it's all good."* ***"****I had to move away. I still know too many people in Perpignan. I was followed by the CSAPA and even there I can't go there anymore because I know the dealers and everything, I've cut off all contact."* **Interview 4:** *"now that I am healed for my family that is the most important thing, for me it doesn't especially change my life, I don't see too many differences, but it changes the lives of those around me."* **Interview 5:** *"I've really cut off this whole environment around me"* **Interview 6:** *"Positive life change is stopping taking drugs, but it's still hard to stop taking them because you think about it, now much less, but stopping isn't hard, it's stopping thinking about it that's hard."* **Interview 8:** *"The best thing about this treatment was that I was able to heal."* **Interview 9:** *"I have a 12-year-old son, I would like to see him grow up." "I haven't touched drugs for 15 years now. Apart from the occasional joint, but otherwise, I don't touch hard drugs anymore, not at all."* **Interview 11:** *"I have little children, you see, and that's why I wanted to get well."* **Interview 12:** *"I have three children, if I was injured, if I was treating them, if they were injured, I could have passed it on to them, so it's something that was important to me. As soon as I was tested, I immediately took the treatment."* **Interview 13:** *"I stopped taking drugs after that."* **Interview 14:** *"I've stopped taking drugs, I've managed to find a place to live, I've got a girlfriend, I've got a life that's a bit more decent, so to speak. All I need is a job and I'm good to go." "I don't associate with the same people anymore, it's not really negative, it's positive, I don't associate with drug addicts anymore."*

Table S2- Qualitative Health Care Professional interviews- mains themes and quotes.

**Reported Intervention limitations**
**COVID-19**"I think overall, we've managed to stay on course despite the difficulties of the pandemic. We can always. We always have the impression that we are not doing enough, that we are not doing enough and that we would like to do more. But in the end, we really closed the activity, the project, for six weeks into the first lockdown. But there, during the second wave, the third wave, we were able to maintain and limit the effects. In the end, this project, with all the mobile part, made it possible to limit the effects of the pandemic on the treatment of viral hepatitis in these vulnerable populations. Because in other regions, this was completely stopped because of the closure of hospitals. We were able to maintain an external activity even when the hospital had closed its doors." [Participant #5]<br>**Choice of community structures** "I think that we have to be careful not to move too far away from our target public when we formalise partnerships. We really have to take care of that. I think that: when we enter into a partnership. That said, it's a personal feeling. Most patients in this structure, in the structures, in the structures where we are going to formalise partnerships must contain our target public rather than an activity for the general population." [Participant # 3]<br>**Weakness in the capacity to do POCT in community structures-** "And about this, the mobile team have noticed that are some difficulties and barriers in the structures." [Participant # 3]<br>"We compensated, we compensated. We are there as a support, in fact, when we are on a Test to Treat session. Of course, we screen, but that was not necessarily our primary mission. We can then come in as a support, but we can't be doing the whole screening in all the structures. That's how the project was set up, in any case. There is a real potential for improvement." [Participant # 3] " [Participant #5] "To do the POCT and there is a decree, in these structures you can't do it like that. They can't just buy the equipment and then do it. No. There's a training, that is to say, framed by a legal decree. And that coordination, perhaps coordination or in any case the regional health agencies, is something that needs to be implemented. It doesn't happen overnight, and it has to be improved." [Participant #5] For example, last week, I trained 24 people, 24 people who can do POCT in their facilities, that are going to start screening in their facilities. There is now a real commitment on the part of the Regional Health Agency, who is trying to implement this training. And also, coordination that has been set up. But it still takes a little bit of time. It is not efficient. It's not efficient at present. [Participant #3] "First of all, in those structures that take care of vulnerable populations, there is a high turnover of staff." [Participant #5]<br>**Pre existing interventions** "Today, it's true that if we observe that the number of patients affected has decreased significantly in the Pyrénées-Orientales, it's because there has been a real effort to adhere to and optimise the work on care, particularly for hepatitis." [Participant # 3]
**Reported alternative models of care and replicability**

MHU replicability

"No. It wouldn't be suitable because of the size of the country. And then the difference, of the difference in practice in different places. Because I think one of the secrets of the success of the mobile team since its inception, is the link with the partners. And everything depends. To discuss this, we had exchange sessions with different teams, from different places in France. In fact, local resources is what counts and the local motivation of partners? You can't copy and paste the team." [Participant # 5] "There's a whole network that exists, but in this case, it really has to be integrated so that there are agreements. For example, with a mobile street team which commits itself to the fact that, when, and this is possible, this is part of their mission and they are financed for that. But when a mobile street team exists on the territory, we also have one, we can very well call them and say "Can you go get Paul and bring him back? So, pick him up at point A and bring him back to me at point B? please, accompany him and everything". [Participant # 3] "Yes, it's true. You can say that technology has reduced the processing time of biological products. As a reminder, in the beginning, the first sessions were done in the hospital via laboratory sampling via a specific organisation. It was possible to be a cheaper model than buying all the equipment. But it is true that adherence and compliance with treatment recorded in this public have, once again, forced towards management with added support." [Participant # 2]

Staff replicability

"there are already nurses today, who are, each expert service, I think, has a budget, the expert services to fight against viral hepatitis which are spread on all the department, on all France, have a budget to carry out outreach activities In fact they have a coordination nurse, it's always like that. They all have a coordinating nurse who is attached to a gastro service as well, and therefore a referring doctor." [Participant # 3]- We've been saying for years that, our team and some others, we have been saying for years that we need to put resources for "outreach" and here, the resources for outreach were really put in place from 2020 onwards. I think it was [nurse] who spoke about it earlier. The funds must be, there were additional funds that were earmarked precisely for this. We have to do outreach. The problem is that, like with many earmarked funds in hospitals, it gets lost a bit, by the directorates which recover, which recover funds for other things. And because some of the teams of these experts do not yet have a culture of "outreach" and are starting slowly. Some have started by opening consultations in prisons in 2019-2020. That's it. They are still involved in actions that we set up in 2013, or even earlier. So, there is still, it's lagging behind. It's definitely very behind." [Participant # 3]

Peer support

"It's not a practice that we use a lot in France. We tried to train peers for therapeutic education and if they were trained, afterwards, we didn't have much therapeutic participation. So, I think it's more because we lack motivated people to make this transition, because it's true that it exists in France, but in what we call self-support associations, that is to say an association that is only a peer association." [Participant # 5]

NGO based model- *"We can very well imagine that mobile teams could be set up from an associative structure (NGO). In this case, there is an association of patients fighting against hepatitis C which looks after this, but I don't know. They need to have found a link with the doctor." [Participant # 5]*
